# Brief report: Assessment of mucosal barrier integrity using serological biomarkers in preclinical stages of rheumatoid arthritis

**DOI:** 10.3389/fimmu.2023.1117742

**Published:** 2023-02-16

**Authors:** Benoît Thomas P. Gilbert, Céline Lamacchia, Lena Amend, Till Strowig, Emiliana Rodriguez, Gaby Palmer, Axel Finckh

**Affiliations:** ^1^ Division of Rheumatology, Department of Medicine, Geneva University Hospitals, Geneva, Switzerland; ^2^ Geneva Centre for Inflammation Research (GCIR), Faculty of Medicine, University of Geneva, Geneva, Switzerland; ^3^ Department of Microbial Immune Regulation, Helmholtz Centre for Infection Research, Braunschweig, Germany; ^4^ Cluster of Excellence Resolving Infection Susceptibility (RESIST) (EXC 2155), Hannover Medical School, Hannover, Germany; ^5^ Center for Individualized Infection Medicine (CiiM), Hannover, Germany; ^6^ Department of Pathology and Immunology, Faculty of Medicine, University of Geneva, Geneva, Switzerland

**Keywords:** rheumatoid arthritis, gut permeability, intestinal inflammation, autoimmunity, biomarker

## Abstract

**Background:**

The pathogenesis of rheumatoid arthritis (RA) is believed to initiate at mucosal sites. The so-called ‘mucosal origin hypothesis of RA’ postulates an increased intestinal permeability before disease onset. Several biomarkers, including lipopolysaccharide binding protein (LBP) and intestinal fatty acid binding protein (I-FABP), have been proposed to reflect gut mucosa permeability and integrity, while serum calprotectin is a new inflammation marker proposed in RA.

**Methods:**

We analyzed serum samples of individuals genetically at increased risk of RA in a nested-case-control study. Participants from a longitudinal cohort of first-degree relatives of RA patients (SCREEN-RA cohort) were divided into three pre-clinical stages of RA, based on the presence of risk factors for subsequent RA onset: 1) low-risk healthy asymptomatic controls; 2) intermediate-risk individuals without symptoms, but with RA-associated auto-immunity; 3) high-risk individuals with clinically suspect arthralgias. Five patients with newly diagnosed RA were also sampled. Serum LBP, I-FABP and calprotectin were measured using commercially available ELISA kits.

**Results:**

We included 180 individuals genetically at increased risk for RA: 84 asymptomatic controls, 53 individuals with RA-associated autoimmunity and 38 high risk individuals. Serum LBP, I-FAPB or calprotectin concentrations did not differ between individuals in different pre-clinical stages of RA.

**Conclusion:**

Based on the serum biomarkers LBP, I-FABP and calprotectin, we could not detect any evidence for intestinal injury in pre-clinical stages of RA.

## 1 Introduction

Rheumatoid arthritis (RA) is an auto-immune disease leading to joint destruction and extra-articular manifestations. Researchers have hypothesized that RA autoimmunity is initially triggered at the mucosal level, for instance in the oral cavity or gastro-intestinal tract (1, 2). In particular, the breakdown of intestinal mucosal barrier integrity and translocation of bacterial products to the circulation and lymphoid organs could constitute a key step (3).

Tajik et al. have first demonstrated in a collagen induced arthritis mouse model how intestinal inflammation and loss of permeability surprisingly precedes the onset of arthritis. Targeting intestinal permeability, using butyrate or zonulin antagonist, reduced the severity of the observed arthritis (4). Using two different mouse models, Matei et al. have confirmed such findings, showing a loss of intestinal integrity before arthritis development. This included epithelial erosion, crypt elongation, reduced expression of tight junction protein 1, translocation of bacterial products to serum and lymphoid organs, and increased serum lipopolysaccharide (LPS) and lipopolysaccharide binding protein (LBP) levels. These observations depended on the presence of gut microbiota, and modification of the intestinal permeability also affected arthritis severity (5). Still, the exact molecular mechanisms linking translocated bacterial products to arthritis remain unclear.

Assessing gut barrier function in humans is challenging. Standard measures of gut mucosal barrier permeability are indirect and rely on the ingestion of passively absorbed probes, most commonly lactulose and mannitol, for which the excreted quantity can subsequently be measured in the urine. A higher urinary lactulose/mannitol ratio (LMR) is believed to indicate a higher small intestine permeability (6). Such functional tests of gut permeability are logistically complicated, time-consuming, and can be compromised by concomitant intestinal disease or NSAID intake (6–8).

To simplify the assessment of gut mucosal barrier integrity, several circulating biomarkers, such as lipopolysaccharide binding protein (LBP), intestinal fatty acid binding protein (I-FABP) and zonulin, have been proposed, even though their reliability in this context is still debated (9). LBP is mostly secreted by the liver and can opsonize gram negative bacteria (10). It also binds circulating LPS, thereby allowing the formation of a ternary complex with CD14 (11) and signaling through TLR4 to induce antibacterial responses (12). Given the technical limitations that prevent direct serum LPS assessment (13), elevated serum LBP levels are sometimes considered to reflect chronic LPS translocation from the intestinal lumen to the circulation (14–18). Serum LBP has also been studied as a marker of inflammation and disease activity in RA patients (19), was reported to modestly correlate with RF titers (20, 21), but has never been assessed during pre-clinical stages of the disease. I-FABP, also known as fatty acid binding protein 2 (FABP-2), is a tissue specific intracellular protein only expressed in enterocytes (22). It is released into the peripheral circulation after epithelial cell injury and is thus used as a marker of intestinal damage, for instance during small bowel ischemia (23), in obesity (24), or in the context of active RA (5).

In the 90’, several authors have evidenced that established RA patients have an increased intestinal absorption of orally administered probes, such as polyethylene glycol, or milk beta-lactoglobulin (7, 25, 26). However, if compromised intestinal mucosal integrity plays a role in the human RA pathogenesis, one would expect mucosal permeability to be altered prior to disease onset, during pre-clinical or early stages of RA (27). Pre-clinical stages of RA are defined by an increased risk for RA development based on genetic or environmental risk factors, the presence of circulating auto-antibodies, or inaugural articular symptoms (27). Only very few studies have assessed intestinal integrity during pre-clinical stages of RA. In their research, Tajik et al. showed elevated serum zonulin in 32 individuals positive for anti-cyclic citrullinated peptide (CCP) auto-antibodies compared to healthy seronegative individuals (5), but using a Cusabio ELISA kit with low reliability (28). Matei et al. assessed serum biomarkers for intestinal integrity in only 7 asymptomatic individuals with RA-related autoimmunity and 7 patients with early stage undifferentiated arthritis. They found no difference in serum I-FABP concentration compared to healthy controls, but slightly elevated serum LPS and LBP concentrations (5).

In this study, we assessed intestinal mucosal barrier integrity in individuals at risk for RA, using LBP and I-FABP as serological surrogate markers. We reasoned that improved feasibility compared to complex oral functional tests would increase participation, and compensate the loss of precision.

Finally, we also assessed serum calprotectin. Calprotectin is a heterodimer of zinc and calcium binding proteins S100 A8 and A9, released by activated macrophages, granulocytes and monocytes. It has a bactericidal effect and promotes inflammatory responses. Serological calprotectin is currently being studied as a promising biomarker for RA disease activity (29–31). Baseline levels at RA diagnosis can predict erosive damage (32, 33) and response to methotrexate (33), while better reflecting disease activity than acute phase proteins (34). Recently, Bettner et al. have demonstrated that serum calprotectin was also elevated in a subset of individuals prior the onset of RA. Combined with RF and ACPA serologies, it improved predictive positive value for future RA diagnosis (35).

## 2 Materials and methods

### 2.1 Study design and population

We analyzed a set of serum samples obtained from the SCREEN-RA cohort, a cohort that follows individuals genetically at risk for RA, namely first-degree relatives (FDR) of established RA patients. This cohort has been extensively described elsewhere (36). Briefly, blood samples are collected at inclusion for genetic testing of the human-leucocyte antigen (HLA), in particular to detect “shared epitope” alleles. The “shared epitope” refers to a group of alleles of the HLA, which strongly increases the risk of RA in case of homozygosity. Serum samples are divided into aliquots, some of which are used for assessment of RA-associated autoantibodies (anti-citrullinated protein antibodies, ACPA; rheumatoid factor, RF; and anti-Ra-33 in a subset of participants) with previously proposed cutoffs (36). The remaining serum aliquots are stored at −80°C. High risk participants for RA, namely participants with auto-immunity associated with RA (i.e. ACPA, RF or anti-Ra33), high genetic risk based on HLA alleles or articular symptoms are followed-up closely and provide new blood samples yearly. All participants also undergo an articular examination at each visit, and complete yearly online questionnaires about lifestyle habits and medical history.

**Figure 1:**
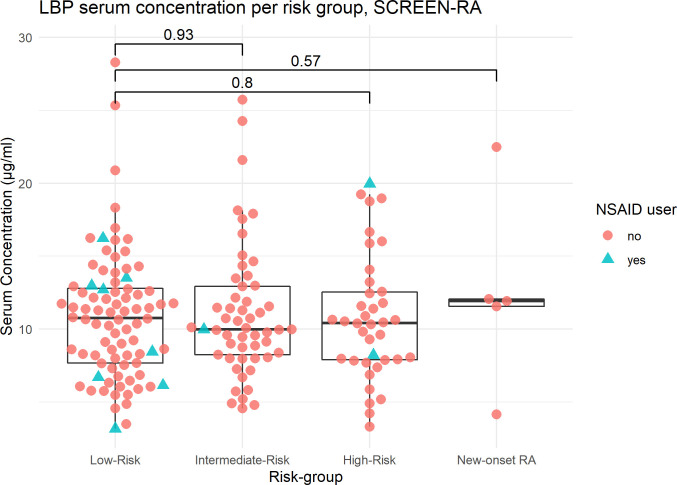
LBP serum concentration per risk subgroup, SCREEN-RA. Serum concentrations of LBP, in: – Low-risk: asymptomatic seronegative FDR of RA patients. – Intermediate-risk asymptomatic FDR with autoimmunity (ACPA, RF, or Ra33). – High-risk FDR with clinically suspect arthralgia, based on a combination of EULAR criteria. – New-onset untreated RA patients. RA, Rheumatoid Arthritis. Outliers are included in the analysis. p-values are displayed (Wilcoxon test). NSAID, NonSteroidal Anti-Inflammatory Drug.

We performed a nested case-control study in a subset of the SCREEN-RA participants (**Figure S1**). Participants were divided into three groups, at sampling timepoint, using combinations of the EULAR proposed terminology for pre-clinical stages of RA (27), which are believed to reflect increasing risk of future RA development (**Figure 1**):

1. Low risk: asymptomatic individuals without RA-specific autoimmunity and without high genetic risk.
2. Intermediate risk: asymptomatic individuals, but with RA-specific auto-immunity, defined as the presence of either RF or anti-Ra-33 antibodies at ≥ 3 times the upper limit of the norm, or the presence of ACPAs at least at the upper limit of the norm.
3. High risk: symptomatic individuals, defined as fulfilling at least 3 of the EULAR criteria for clinically suspect arthralgia (37), regardless of serological or genetic status.

We invited available intermediate and high-risk participants to provide a new blood sample between 2019 and 2021. In parallel, low-risk participants, who are in excess in the cohort, were selected so that the age and sex ratio were comparable to the two other groups, and invited likewise for sampling. Consecutive patients with untreated new-onset RA were also recruited from the rheumatology division of Geneva University Hospital, during the study period.

We performed a sensitivity analysis taking into account the longitudinal evolution of these individuals during an average follow-up of 1.76 years. Depending on the individual’s change in symptoms or signs of the disease, we categorized participants as ‘progressors’, ‘regressors, or ‘stable’ and analyzed the correlations with the biomarkers of interest.

### 2.2 Sample analysis

We used commercially available sandwich DuoSet ELISA kits from R&D Systems (Minneapolis, MN), for LBP (DY870, range 0.78 – 50 ng/ml), calprotectin (DY1820, range 93 - 6000 pg/ml) and I-FABP (DY3078, range 31 - 2000 pg/ml). Samples were randomized and divided into three batches. Each batch was aliquoted in several 96-wells plates, at the appropriate dilution. Then, for a given marker to be tested, ELISA tests were run in duplicate, during 3 consecutive days, according to the manufacturer’s instructions (**Figure S2**). For the LBP and calprotectin assays, samples were diluted 1/1000, while for the I-FABP assay, samples were diluted 1/10, in reagent diluent. Due to the preparation procedure, all samples were thawed twice before measurement (i.e. initial freezing, thawing, dilution and aliquoting, re-freezing, final thawing and testing).

Optical density was determined using a LEDETECT 96 automatic reader, set to 450 nm with a correction filter at 570 nm. Finally, for each plate, the standard curve was constructed with R code using the *drm* function from the *drc* package v.3.0-1 to convert optical densities into concentration values. For each duplicated measurement, the inter-assay coefficient of variation of the two optical densities was computed as (standard deviation)/(mean). Only samples with <10% CV were included in the final analysis. The marker concentration was obtained by averaging the two measured concentrations, and multiplying by the dilution factor.

### 2.3 Statistical analysis

For baseline characteristics, continuous variables are expressed as means and standard deviations (SD), while categorical variables are expressed using percentages. ANOVA, χ2 test or Fisher’s exact test for small size samples were used to compare baseline characteristics between groups.

The biomarker concentrations were compared to the low-risk group using two-sided Wilcoxon rank tests. Correlations between the biomarkers were calculated using Spearman coefficient, with the related p-value. All statistics were performed using R 2022.02.3 with package *tableone* and *stats*.

## 3 Results

Out of the 1539 participants of the SCREEN-RA study, we selected 180 individuals, matching low-risk with intermediate- and high-risk participants for sex and age. This resulted in: 84 low-risk individuals, 53 intermediate-risk individuals, and 38 high-risk individuals. Five untreated new-onset RA patients were also recruited, and sampled at the time of RA diagnosis, prior to antirheumatic treatment initiation. There were no significant differences between the groups in terms of age, gender and BMI (**Table 1**).

**Table 1:** Baseline characteristics.

Variable	Groups			
	Low-risk n = 84	Intermediate- risk n = 53	High-risk n = 38	New-Onset RA n = 5
Female gender	81%	75%	87%	100%
Age mean (SD)	54 (13)	54 (16)	55 (11)	53 (17)
BMI mean (SD)	25 (5)	24 (4)	24 (5)	26 (6)
ACPA positivity (>1x norm)	0%	32%	8%	60%
RF positivity				
1 to 3x the norm >3x the norm	11% 0%	8% 66%	16% 10%	0% 60%
Anti-Ra33 positivity				
1 to 3x the norm >3x the norm	13% 0%	21% 4%	13% 0%	NA
With detectable RA auto-immunity (low threshold >1x norm)	20%	100%	42%	60%
Shared epitope alleles				
0 allele 1 allele 2 alleles	48% 52% 0%	49% 38% 13%	55% 39% 7%	40% 40% 20%

ACPA, Anti-Citrullinated Protein Antibodies. RF, Rheumatoid Factor. Anti-Ra33, anti-Ra33 autoantibodies. CSA, Clinical Suspect Arthralgia according to the EULAR definition.

Low risk = asymptomatic RA-FDR without specific RA- autoimmunity. Intermediate risk = asymptomatic RA-FDR with specific RA-autoimmunity. High risk = symptomatic RA-FDR. New-onset RA are untreated at sampling time. NA, Not Assigned, i.e. the new onset RA were not tested for anti-Ra33 antibodies.

**Figure 2:**
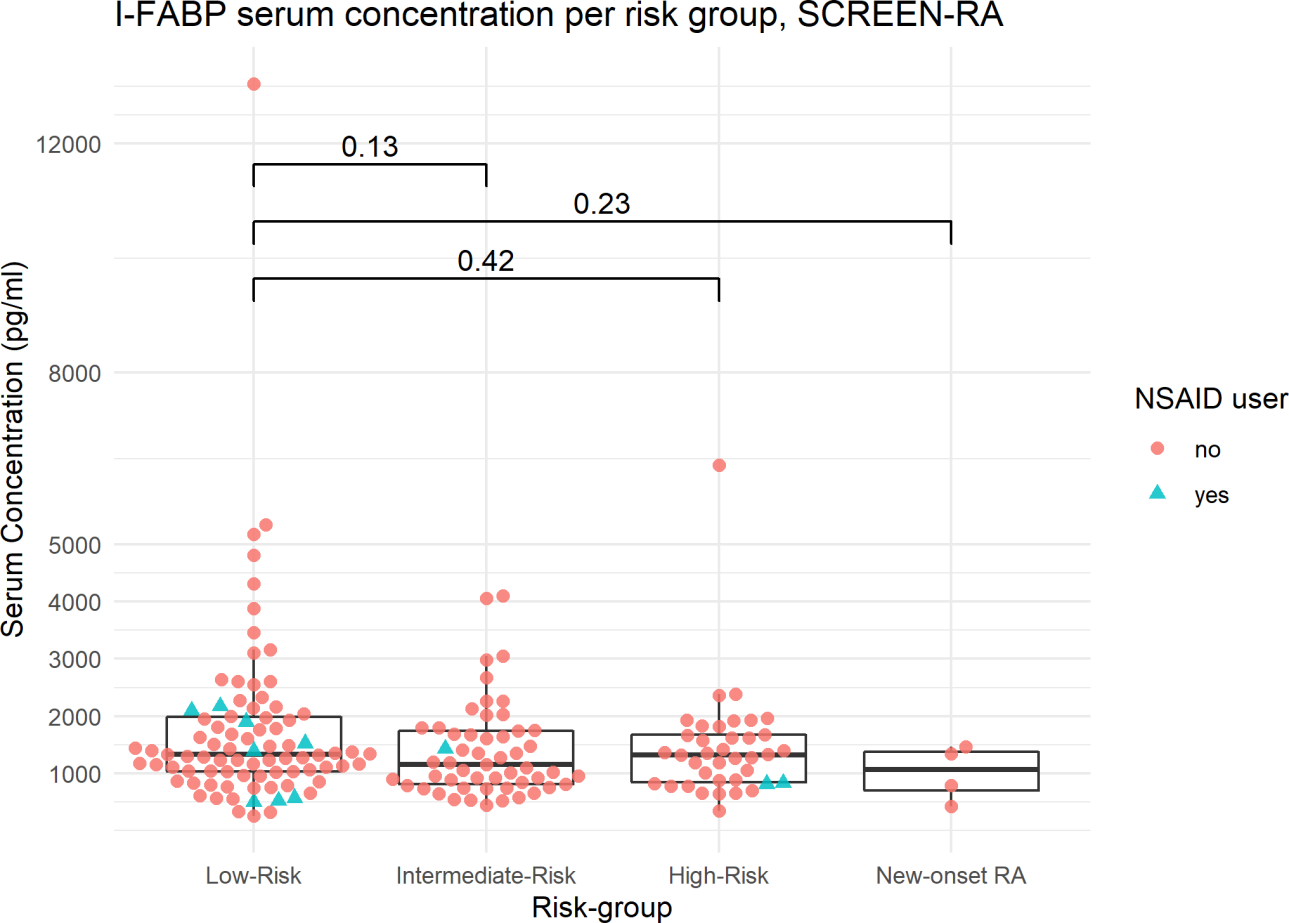
I-FABP serum concentration per risk subgroup, SCREEN-RA. Serum concentrations of I-FABP, in: – Low-risk: asymptomatic seronegative FDR of RA patients. – Intermediate-risk asymptomatic FDR with autoimmunity (ACPA, RF, or Ra33). – High-risk FDR with clinically suspect arthralgia, based on EULAR criteria. – New-onset untreated RA patients. RA, Rheumatoid Arthritis. Outliers are included in the analysis. p-values are displayed (Wilcoxon test). NSAID, NonSteroidal Anti-Inflammatory Drug.

LBP, I-FABP and calprotectin concentrations were assessed in the serum of all participants. The mean inter-assay coefficient of variation (CV), computed on optical densities, was 1.7% for LBP, 2.2% for I-FABP, and 3% for calprotectin. One sample was excluded from the I-FABP analysis, and 7 samples were excluded from the calprotectin analysis, because the difference between the two replicates was too large (CV >10%). Overall, the mean values of the three biomarkers did not differ between the groups (**Figures 1**, **2** and **S3**; **Table 2**). Outliers were kept in the analysis.

**Table 2:** Biomarker concentrations.

Variable	Groups			
	Low-risk n = 84	Intermediate-risk n = 53	High-risk n = 38	New-onset RA n = 5
Number of samples analyzed, n LBP, n I-FABP, n Calprotectin, n	84 84 82	53 53 50	38 38 36	5 4 5
LBP (μg/ml), mean (SD)	10.83 (4.39)	11.07 (4.55)	11.75 (4.27)	12.44 (6.53)
I-FABP (pg/ml), mean (SD)	1746 (1617)	1393 (823)	1438 (965)	1009 (487)
Calprotectin (ng/ml), mean (SD)	2043 (1396)	1860 (1163)	1629 (1114)	1897 (649)

LBP, Lipopolysaccharide Binding Protein; I-FABP, Intestinal Fatty Acid Binding Protein; SD, Standard Deviation.

We found no correlation between LBP and I-FABP levels (Spearman rho -0.06; p = 0.40), nor between I-FABP and calprotectin serum concentrations (Spearman rho -0.07; p = 0. 36; **Figure S4**). LBP modestly correlated with systemic inflammation, as reflected by serum calprotectin levels (Spearman rho = 0.32; p < 0.001; **Figure S3**), but not with RF status (data not shown). In complementary experiments, we noticed that additional thawing cycles reduced detectable protein concentrations for LBP, but not for I-FABP and calprotectin (**Figure S4**).

Finally, follow-up data was assessed in January 2023. Average time-difference between serum sampling and last news date was 1.76 years (SD = 0.74). In terms of risk group attribution, 16 individuals progressed, 32 individuals regressed, and 132 remained in the same risk group. Of note, one patient, enrolled in the low-risk group, developed RA 25 months after serum sampling (measured LBP = 25.34 μg/ml; I-FABP = 1025 pg/ml). We found no evidence of higher serological concentration of I-FABP or LBP associated with progression toward auto-immunity or clinically suspect arthralgia during this time-frame (**Figure S6**).

## 4 Discussion

It has been evidenced in several mouse models that compromised intestinal integrity and increased translocation of bacterial compounds precede and affect the onset of arthritis (4, 5). In this work, we measured a panel of three biomarkers, which have been proposed to reflect respectively exposure to LPS translocation (LBP), intestinal integrity (I-FABP) and systemic inflammation (serum calprotectin), in the serum of individuals in preclinical stages of RA. Given the uncertainty concerning commercially available zonulin ELISA tests, we did not assess zonulin. Indeed, zonulin ELISA kits also target related (38) and unrelated peptides, such as properdin, which belongs to the zonulin family, and complement C3 (28). It is consequently still unclear what the commercially available zonulin tests actually detect (39).

Unexpectedly, we observed no differences in LBP and I-FABP between our groups of interest. One potential hypothesis explaining the negative findings is that our assessments were performed too late, or too early, in the timeframe of disease development. Indeed, our intermediate- and high-risk groups were already displaying auto-immunity or symptoms for several months or years, while still being a long time before possible arthritis onset.

Several considerations should also be discussed regarding the use of biomarkers to assess intestinal integrity.

First, we could not find a consensual definition of normal serum LBP levels. Mean reported values in healthy control groups range between 5 and 19 ug/ml (5, 40–47). In addition to natural inter-subject variability, the observed variations in these control concentrations might reflect different handling procedures, different dilutions, and different numbers of thawing cycles before measurement. The latter is rarely reported, and we observed that one additional sample freezing step reduces the measured concentration of LBP by approximately twofold (**Figure S5**), which could partly explain conflicting results. Also, it has to be kept in mind that RF can sometimes interfere with immunoassays, inducing falsely positive results (48).

After carefully re-considering the literature, we feel that previous findings regarding LBP should be interpreted with caution. It has been known since the 90’ that LBP induction in the liver depends on IL-1β and IL-6, which makes it an acute-phase protein (49), although extra hepatic secretion by adipocytes has also been documented (50). In the context of RA, this could explain why LBP correlates with disease activity markers, such as erythrocyte sedimentation rate and C-reactive protein (CRP) (5, 19, 51). On the other hand, LPS is by itself a strong pro-inflammatory agent, which might contribute to low-grade systemic inflammation, adding even more confusion to the matter (13). Thus, it is not clear whether increased LBP is to be seen as the result of systemic inflammation, LPS-induced endotoxemia, or both. In our study, we noticed a modest correlation between LBP and serum calprotectin, while LBP serum concentrations did not differ between the three studied groups. Similarly, Matei et al. were not able to distinguish healthy controls from individuals in pre-clinical stages of RA using LBP (5).

Reported mean I-FABP values in healthy individuals range from ~300 pg/ml to ~1300 pg/ml, depending on the ELISA methodologies and suppliers (5, 52–54). Factors potentially confounding I-FABP serum level include intensive exercise (52, 55) and NSAID intake (56). Matei et al. have shown I-FABP to be significantly elevated in active RA patients, compared to healthy controls (5). This difference was not observed when comparing controls to individuals in the pre-clinical stages of RA, even though for the latter comparison sample size was limited (7 pre-clinical RA versus 34 controls) (5). The latter finding was independent of 2NSAID, NonSteroidal Anti-Inflammatory Drug intake or disease activity. Similarly, we did not observe any differences in serum I-FABP levels between high-, intermediate- and low-risk probands. Only a minority of participants in our study reported NSAID treatment, which did not appear to interfere with our conclusions. Noteworthily, we found no correlation between I-FABP and LBP levels, which was also noticed by Amarrudin et al. in a population of children treated for helminth infection (15).

Serum calprotectin reflects granulocyte activation, and usually does not exceed 1000 – 1500 ng/ml in healthy state (34, 57–59). In the context of acute disease, such as severe COVID-19, or active Crohn’s disease, serum calprotectin can reach 10’000 to 20’000 ng/ml (58, 59). In the present study, we found a modest correlation between LBP and calprotectin serum levels. However, asymptomatic auto-immunity associated with RA or clinically suspect arthralgia did not appear to significantly influence serum calprotectin levels.

### 4.1 Limitations and strengths

The ELISA testing procedure used has several limitations: first, serum samples were thawed twice, which could lead to an underestimation of high marker concentrations, in particular for LBP (**Figure S5**). Also, for technical reasons, samples were divided into 3 different batches, for which ELISAs were run on different days. Even though these three batches were analyzed on three consecutive days with the same procedure, by the same operator, we cannot *a priori* exclude batch effects. We tried to minimize the impact of potential batch effects by randomizing samples across the three batches.

The major limitation of this study is that we did not perform oral-sugar intestinal permeability tests, because of practical considerations. Hence, we cannot formally exclude that the three groups may differ in terms of LMR. Overall, there is only limited evidence that serum biomarkers reflect mucosal barrier permeability or LMR ratios (**Table S1**). Also, recent studies have underlined that plasma should be preferred to serum for calprotectin measurement – thus we may have overestimated calprotectin concentrations due to monocyte and granulocyte release during blood coagulation (60). Finally, NSAID usage was self-reported by online questionnaire. We can thus not exclude that some NSAID users have not documented their treatment. The main strengths of this study are its large sample size, duplicate measurements, and multiple marker dosage in a single serum sample.

Including only FDR individuals ensured comparable genetic background between groups. A drawback is the lack of healthy asymptomatic controls, without family history of RA. However, it is important to underscore that the incidence of RA remains low in FDR of RA patients, with life-time risk of developing RA between 1-2% (61), so that we believe that the risk of RA in asymptomatic first degree relatives is a good proxy for a healthy control population. To our knowledge, it is unclear if genetic risk for RA correlates with baseline levels of serum I-FABP and LBP. Finally, the few untreated new-onset RA that we managed to sample did not allow to constitute a group with sufficient size. Future research would certainly benefit from studying such new-onset RA patients, given that they exhibit a clear phenotype, without the interference of immunosuppressive medications.

### 4.2 Conclusion

We found no association between putative serum biomarkers of intestinal integrity (LBP and I-FABP) and preclinical stages of RA development. Also, serum LBP did not correlate with I-FABP, but correlated with serum calprotectin, which further questions the relevance of LBP as a marker of gut epithelial health. Future research needs to clarify if LBP truly signals changes in the intestinal integrity or instead merely reflects systemic inflammation.

## Data availability statement

The original contributions presented in the study are included in the article/**Supplementary Material**. Further inquiries can be directed to the corresponding author.

## Ethics statement

The studies involving human participants were reviewed and approved by Cantonal Commission on Research Ethics (CCER) of Geneva. The patients/participants provided their written informed consent to participate in this study.

## Author contributions

BG: sampling, sample analysis, data interpretation, article writing. CL: biobanking, data interpretation, article correction. LA: study design, data interpretation, article writing. TS: study design, data interpretation, article correction. ER: protocol optimization, sample analysis, data interpretation. GP: study design, protocol optimization, data interpretation, article correction. AF: study design, supervision, data interpretation, article correction. All authors contributed to the article and approved the submitted version.
