# Supplementary material for: Brief report: Assessment of mucosal barrier integrity using serological biomarkers in preclinical stages of rheumatoid arthritis
Source: Front Immunol. 2023 Feb 16;14:1117742. doi: 10.3389/fimmu.2023.1117742 (PMC9977794; doi:10.3389/fimmu.2023.1117742)
Supplement: Supplementary file 2 [file DataSheet_2.docx]

This dataset has been generated based on serum sampled between 2019 and 2021, frozen at -80°C, and analyzed in 2022 at the University of Geneva.

All individuals are part of the Screen-RA cohort : https://bmjopen.bmj.com/lookup/doi/10.1136/bmjopen-2020-048409

subject_id: unique identifier of enrolled participant (coded).

value_LBP: serum LBP concentration determined by ELISA (ug/ml)

value_IFABP: serum IFABP concentration determined by ELISA (pg/ml)

value_S100: serum calprotectin concentration determined by ELISA (ng/ml)

group: grouping variable (see publication for definition)

medication_tags_imp: catergories of medications mentioned by the patient in his/her closest follow_up form.

stool_consistence: stool consistency at the time of serum sampling - bristol scale score.

ra33: anti-ra33 antibodies serology in a previous serum sample - N = Negative, L = Low (i.e. 1 to 3 times the upper limit of the norm), H = High (i.e. >3 times the upper limit of the norm).

rf: rheumatoid factor antibodies serology at last news - N = Negative, L = Low (i.e. 1 to 3 times the upper limit of the norm), H = High (i.e. >3 times the upper limit of the norm).

acpa: anti-citrullinated peptide antibodies serology at last news - N = Negative, L = Low (i.e. 1 to 3 times the upper limit of the norm), H = High (i.e. >3 times the upper limit of the norm).

autoimmu (yes/no) : tags the patients who had detectable auto-immunity of at least one marker. However, here the used cutoff is 1x the Upper Limit of the Norm. Hence some control indidivuals have low levels of RF or ra33 (which are usually not considered clinically significant).

age: age in years

gender: gender, as a label

bmi: body mass index

X_value_LBP: LBP serum concentration measured in sample after one additional thawing cycle

X_value_IFABP: IFABP serum concentration measured in sample after one additional thawing cycle

X_value_S100: S100 serum concentration measured in sample after one additional thawing cycle

BOX: each marker measurment was done in three batches. This is the bacth number

Empty cells are missing values.
