# Supplementary material for: Brief report: Assessment of mucosal barrier integrity using serological biomarkers in preclinical stages of rheumatoid arthritis
Source: Front Immunol. 2023 Feb 16;14:1117742. doi: 10.3389/fimmu.2023.1117742 (PMC9977794; doi:10.3389/fimmu.2023.1117742)
Supplement: Supplementary file 3 [file DataSheet_3.docx]

| **Table S1 - Literature review of the association between oral permeability tests and surrogate biomarkers** | | | | | | | |
| --- | --- | --- | --- | --- | --- | --- | --- |
| **Context** | **Oral test** | **Zonulin** | **LBP** | **sCD14** | **I-FABP** | **IL-6** | **Ref.** |
| Type 1 diabetes: 36  Relative: 56  Healthy controls: 43 | LMR | **Correlated**  (driven by 4 outliers) | - | - | - | - | Sapone et al. 2006 (56) |
| Crohn disease with (16) or without (12) high GM-CSF Auto-Ab, and healthy controls (15) | LMR | **-** | **Not correlated** | - | - | - | Nylund et al., 2011 (57) |
| Healthy young males: 20  Crossover, several measures | LMR | **Not correlated**  (data not shown) | **-** | - | - | - | Russo et al 2012 (58) |
| Obese: 12  Normal: 12  3x samples (longitudinal) | LMR | **Not correlated** | **Not correlated** | - | - | - | Kuzma et al, 2016 (59,60) |
| Cirrhosis patients: 46  Healthy controls: 16 | LMR | **-** | **Not correlated** | **-** | **Not correlated** | **Correlated**  (modest) | Vogt et al. 2016 (61) |
| Migraine patients: 63 | LMR | **Not correlated**  (data not shown) | **-** | **-** | **-** | **-** | De Roos et al. 2017 (62) |
| Type 2 diabetes: 32  Controls healthy: 30 | 52Cr-EDTA | **-** | **Not correlated** | **Not correlated** | **-** | **-** | Pedersen et al. 2018 (63) |
| Crohn disease patients: 60 | 52Cr-EDTA | **-** | **-** | **-** | **-** | **-** | Von Martels et al. 2019 (64) |
| Human and mice experiments | LMR | **Not reported**  (let’s ask them?) | **-** | **-** | **-** | **-** | Tajik et al. 2020 (4) |
| Healthy: 51  Obese: 27 | LMR | **Not correlated** | **Correlated**  (independent of age, BMI and sex) | **-** | **Not correlated** | - | Seethaler et al. 2021 (37) |
| Children in Indonesia | LMR | **-** | **Not correlated** | **-** | **Not correlated** | **-** | Amaruddin et al. 2022 (15) |
| LMR = urinary Lactulose Mannitol Ratio, dosed after oral challenge. | | | | | | | |

Table S1 reviews the reported associations between oral gut-permeability tests and serum surrogate markers. Sapone et al. have first reported that in the context of type 1 diabetes, increased zonulin level was associated with increased LMR, but this seems to misleadingly rely mostly on 4 outliers (Figure 2C in ref (56) - linear regression should not have been used in this case). Kuzma et al., in a small cohort with longitudinal measures, could not observe any correlation between LMR and fasting LBP.(59,60) Zonulin did not correlate with LMR either. LBP and zonulin measures demonstrated good intra-individual reproducibility.(59,60) Vogt et al., working on cirrhosis, found that neither LBP nor I-FABP serum concentrations were associated with LMR.(61). Similarly, Nylund et al., studying Crohn disease, did not find any association between serum LBP and LMR ratios. Recently, Seethaler et al. opposed previous findings and demonstrated a moderate positive correlation between LMR and serum LBP, both in healthy (Spearman r = 0.42; p= 0.001) and obese individuals (r = 0.78; p = 0.008).(37) But LMR still did not correlate with serum I-FABP, nor with serum zonulin, while LMR increased with obesity.(37)


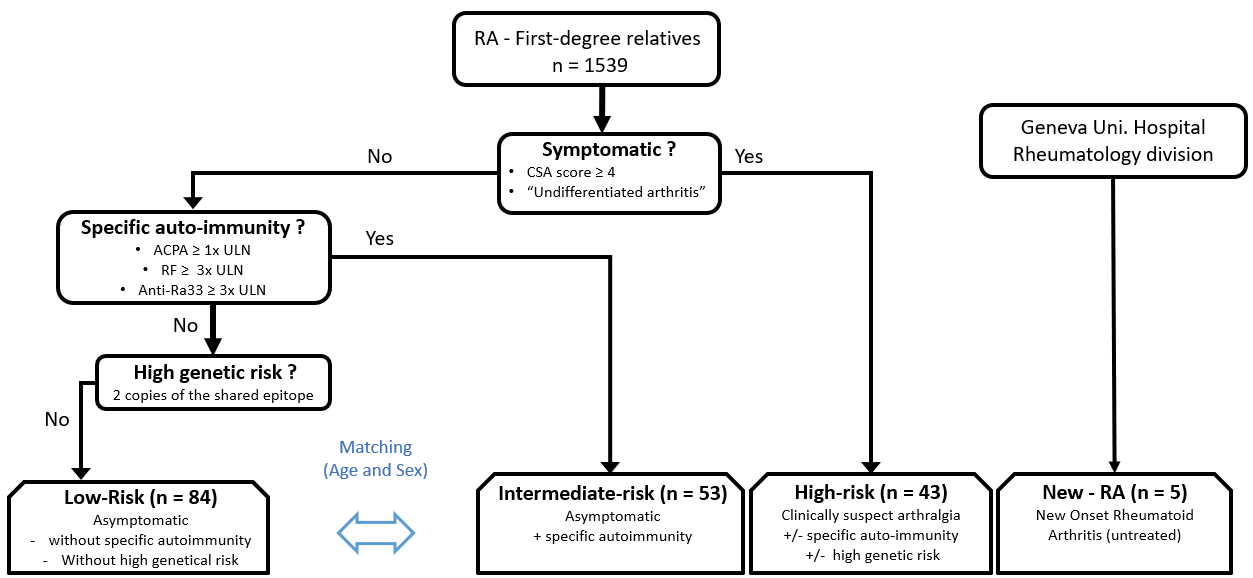


**Figure S1: Selection of included serum-samples**

This figure illustrates the selection process for serum samples. Each participant provided one sample. The low-risk participants, in excess within the cohort, were selected to form a group matched for age and sex to each of the two higher risk groups. RA = Rheumatoid Arthritis. ACPA = Anti-Citrullinated Protein Antibodies. RF = Rheumatoid Factor. Anti-Ra33 = Anti-Ra33 auto-antibodies. ULN = Upper Limit of the Norm. CSA score = Clinically Suspect Arthralgia, based on the EULAR proposition. High-genetic risk individuals were excluded from the Low-risk group.


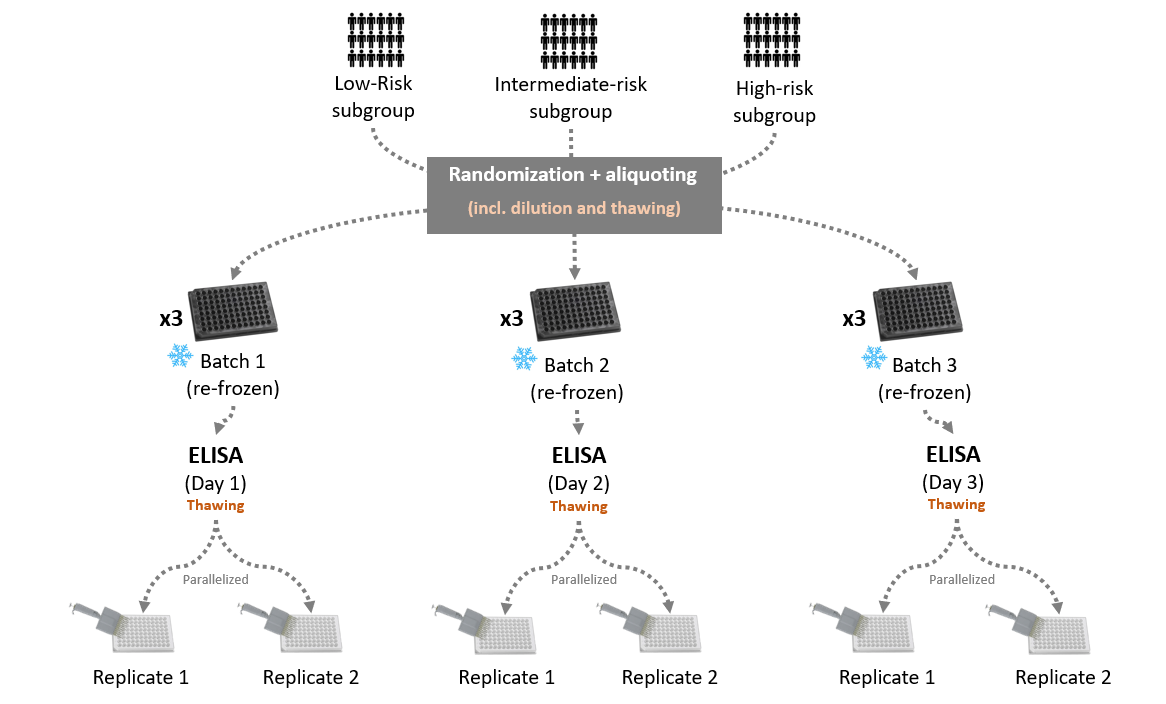


**Figure S2: serum sample processing for ELISA testing**

This figure illustrates how serum samples were processed to measure LBP, I-FABP and calprotectin. Frozen serum samples were first randomized into three batches. For each batch, three plates containing serum aliquots at the required dilution were prepared and re-frozen. Finally, for a given serological marker, a plate of each batch was thawed and ELISA test performed on three consecutive days in duplicate.


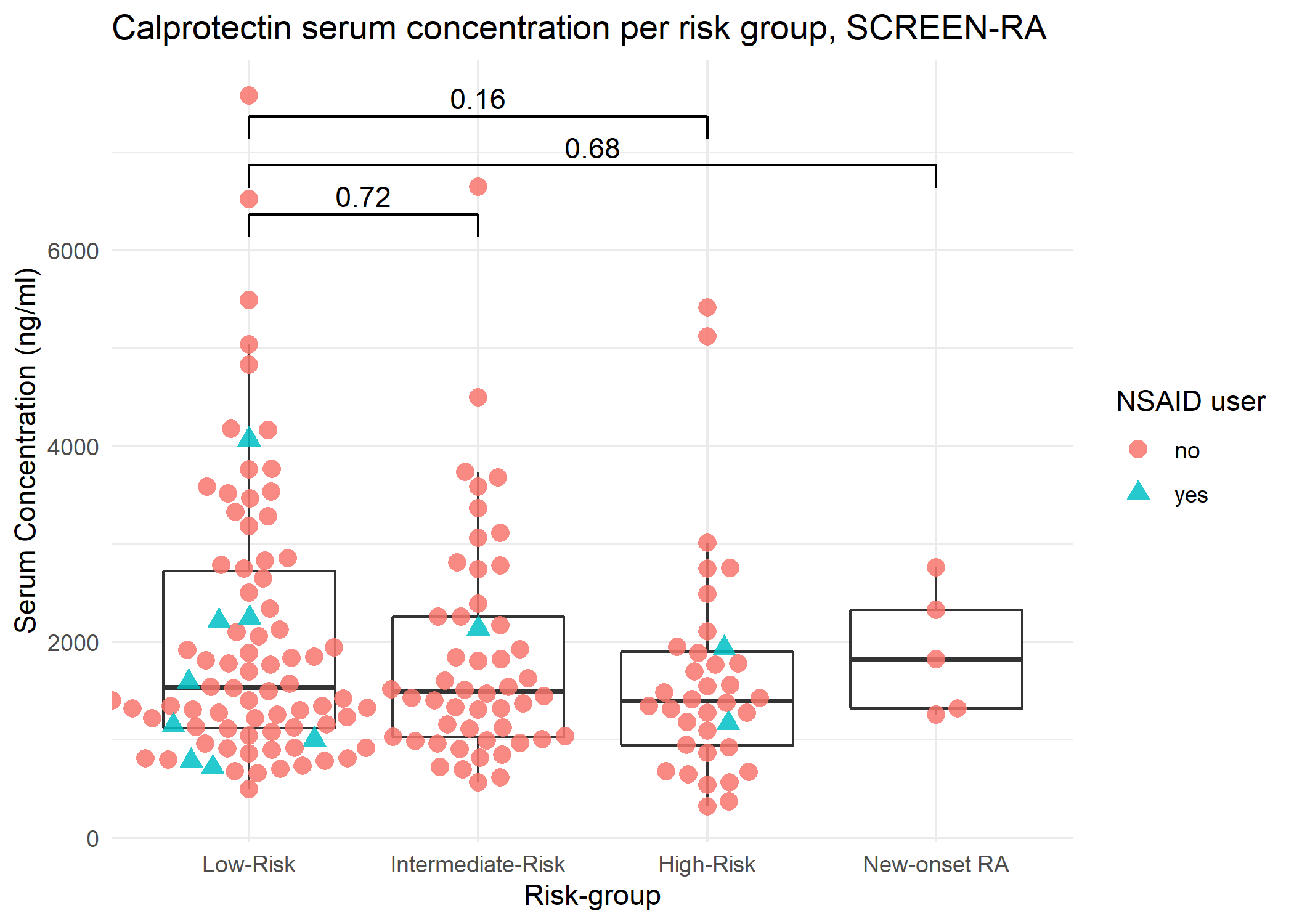


Figure S3: Calprotectin serum concentration per risk subgroup, SCREEN-RA

Serum concentrations of calprotectin, in: – Low-risk: asymptomatic seronegative FDR of RA patients. – Intermediate-risk asymptomatic FDR with autoimmunity (ACPA, RF, or Ra33). – High-risk FDR with clinically suspect arthralgia, based on EULAR definition. – New-onset untreated RA patients. RA: Rheumatoid Arthritis. Outliers are included in the analysis. p-values are displayed (Wilcoxon test).


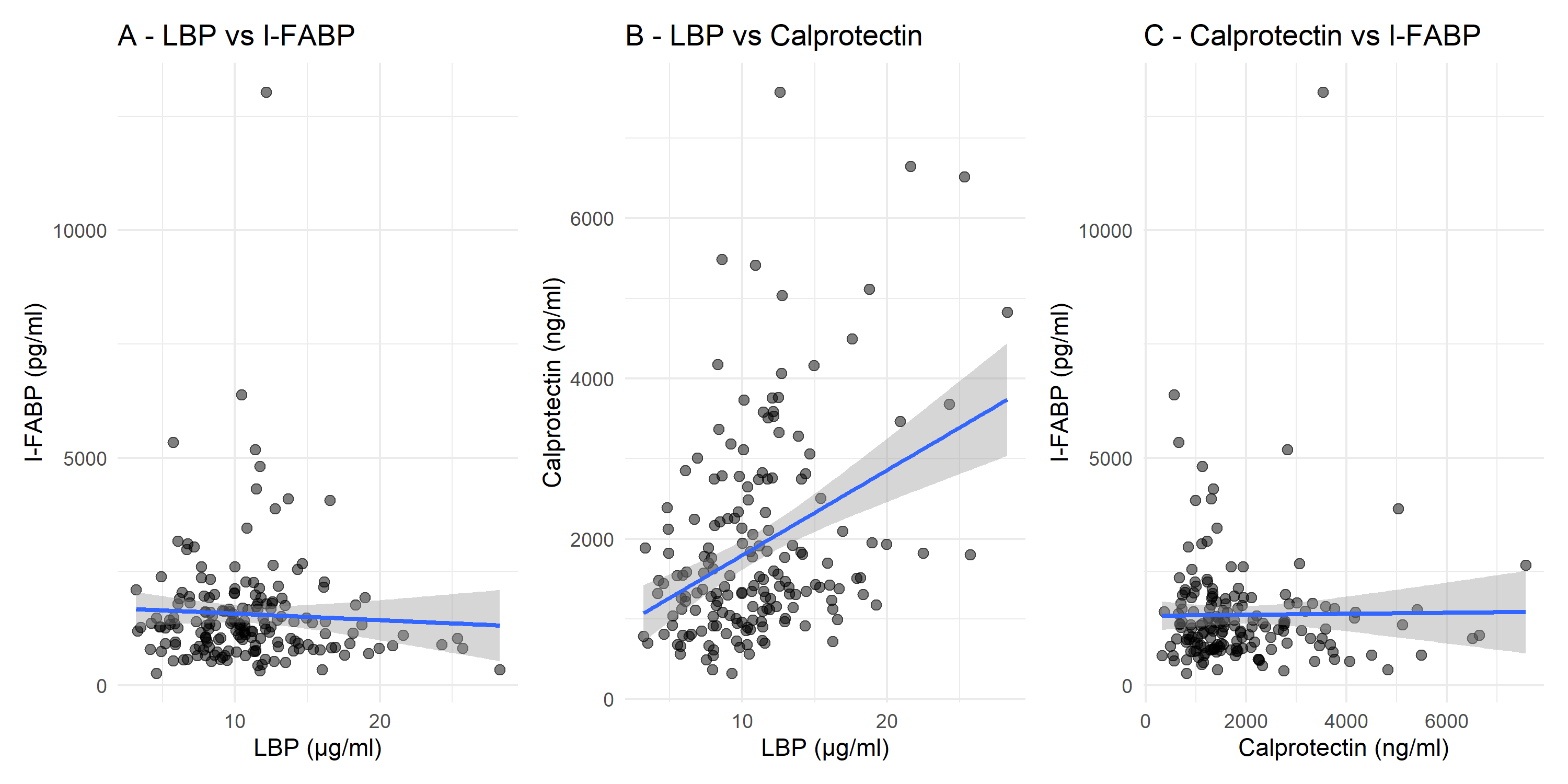


Figure S4: Serum LBP, I-FABP and calprotectin concentrations correlations, SCREEN-RA

Correlation of serum concentrations for LBP, I-FABP and calprotectin. The line is a fitted linear regression model. LBP = Lipopolysaccharide Binding Protein. I-FABP = Intestinal Fatty-Acid Binding Protein. Spearman coefficient for LBP versus calprotectin: rho = 0.32; p < 0.001.


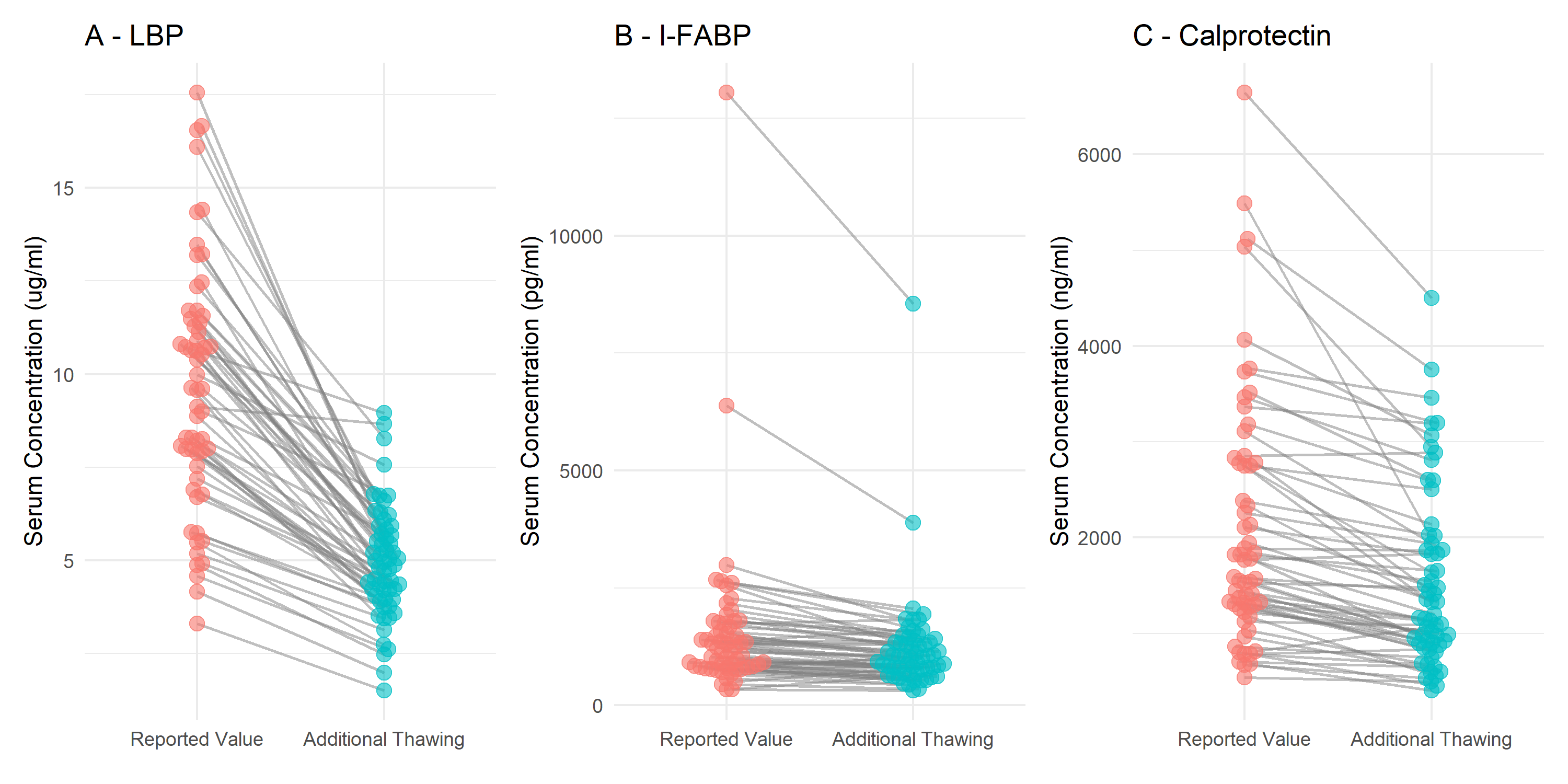


Figure S5: Influence of one additional thawing cycle on ELISA result.

As a quality check, every sample underwent an additional ELISA testing, for one of the three markers, after a third thawing cycle. This later value is compared to the value reported in the main body of the article. Overall, Spearman correlation between the two values were high and significant for every marker (LBP: Spearman rho 0.74, I-FABP: Spearman rho 0.92, Calprotectin: Spearman Rho: 0.93; all p < 0.001). However, LBP seemed more affected by thawing cycles than the two other makers, with mean detectable concentrations divided by ~2.


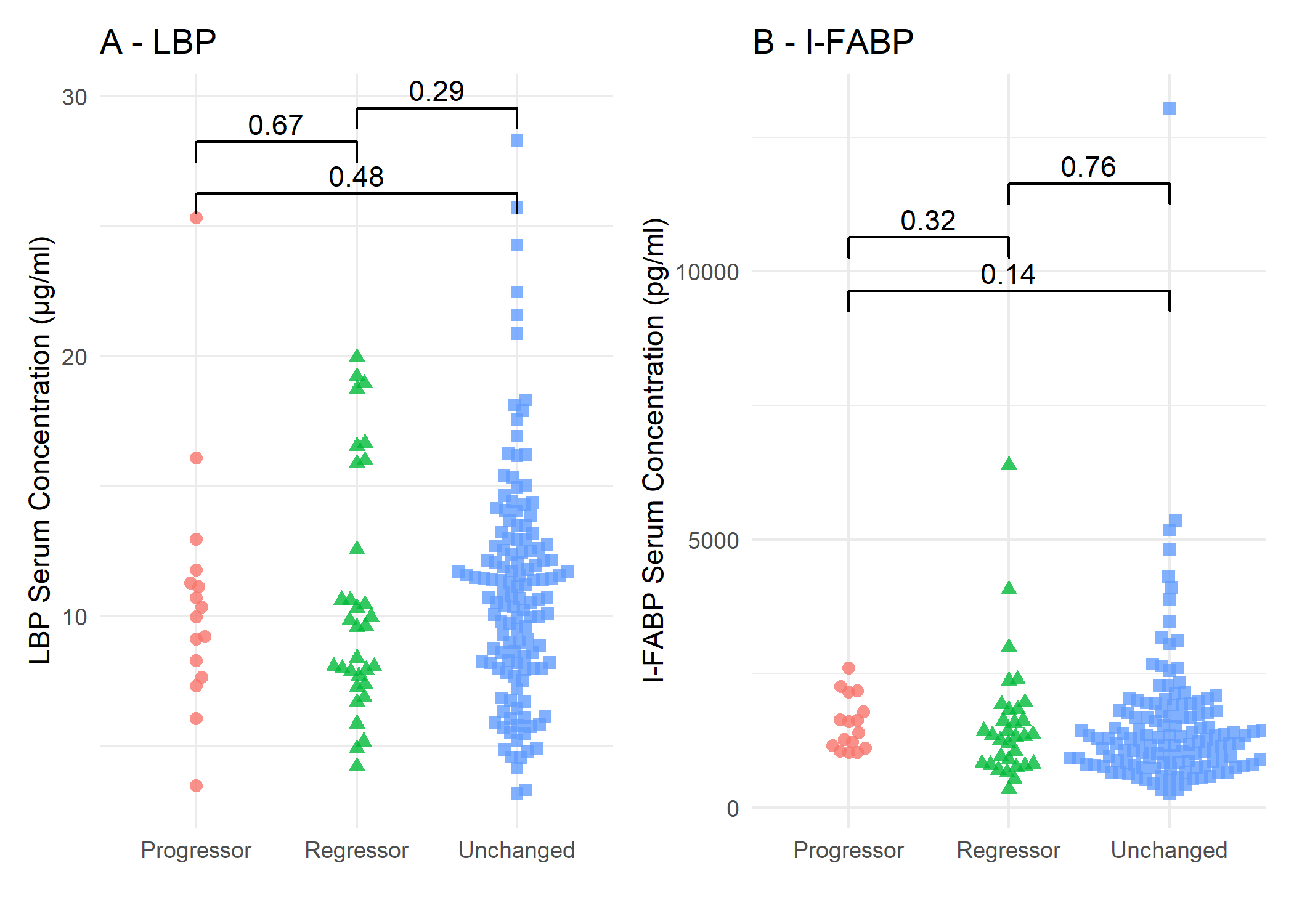


Figure S6: Marker concentration by later follow-up, SCREEN-RA.

Average time-difference between serum sampling and last news date was 1.76 years (SD = 0.74). In terms of risk group attribution, 16 patients progressed, 32 patients regressed, and the rest (132) remained in the same group. Of note, one patient, enrolled in the low-Risk group, developed confirmed RA during follow-up, 2.07 years after serum sampling (measured LBP = 25.34 μg/ml ; I-FABP = 1025 pg/ml).
